# Mechanisms of action for 2-phenylethanol isolated from *Kloeckera apiculata* in control of *Penicillium* molds of citrus fruits

**DOI:** 10.1186/s12866-014-0242-2

**Published:** 2014-09-19

**Authors:** Pu Liu, Yunjiang Cheng, Meng Yang, Yujia Liu, Kai Chen, Chao-an Long, Xiuxin Deng

**Affiliations:** Key Laboratory of Horticultural Plant Biology of the Ministry of Education, National Centre of Citrus Breeding, Huazhong Agricultural University, Wuhan, 430070 P. R. China; Key Laboratory of Pomology, Anhui Agricultural University, Hefei, 230036 P. R. China; Key Laboratory of Genome Sciences and Information, Beijing Institute of Genomics, Chinese Academy of Sciences, Chaoyang District, Beijing, 100029 P. R. China

**Keywords:** Biological control, *Penicillium*, *Kloeckera apiculata*, Antifungal compound, 2-phenylethanol (PEA), Postharvest

## Abstract

**Background:**

Green and blue mold decay, caused by *Penicillium digitatum* and *P. italicum*, respectively, are important postharvest diseases of citrus. Biocontrol by microbes is an alternative to synthetic fungicide application. In this study, the antagonistic yeast strain *Kloeckera apiculata* 34–9 was used to investigate the action mechanisms involved in the biocontrol of postharvest diseases.

**Results:**

An antifungal substance, 2-phenylethanol (PEA), was isolated from *K. apiculata* and demonstrated to have antimicrobial activity against selected phytopathogenic fungi. Experiments on *P. italicum* cells identified the mitochondria and the nucleus as particularly sensitive to inhibition. Regulation of *P. italicum* gene expression was investigated using RNA-Seq. PEA up-regulated genes involved with the peroxisome, regulation of autophagy, phosphatidylinositol signaling system, protein processing in endoplasmic reticulum, fatty acid metabolism, and inhibited ribosome, RNA polymerase, DNA replication, amino acid biosynthesis, aminoacyl-tRNA biosynthesis and cell cycle. Inhibitory responses revealed by RNA-Seq suggest that PEA might compete for attachment on the active site of phenylalanyl-tRNA synthetase (PheRS).

**Conclusion:**

This study provided new insight on the mode of action of biocontrol yeast agents in controlling postharvest pathogenic fungi.

**Electronic supplementary material:**

The online version of this article (doi:10.1186/s12866-014-0242-2) contains supplementary material, which is available to authorized users.

## Background

*Penicillium digitatum* and *P. italicum*, the causal agent of green and blue mold decay, respectively, are important postharvest diseases of citrus and cause heavy losses around the world [1]. Biological control using microbial agents (bacteria, yeast and fungi) is considered to be a viable alternative to the use of synthetic fungicides. Among microbial agents, yeasts have several good properties that make them ideal antagonists, including the ability to survive in adverse environmental conditions, having few nutritional requirements and being amenable to formulation with a long shelf-life [2,3]. Furthermore, yeast strains can degrade mycotoxins, which are well known for being toxic to humans and animals [4]. Over 30 yeasts have been isolated and investigated for their biocontrol efficacy against postharvest fruit diseases. Some yeast-based products have been registered as commercially available biocontrol agents such as “Aspire” (*Candida oleophila* 182; Ecogen, Langhorne, PA, USA) [5]. Knowledge about the modes of action of biocontrol agents is essential for developing appropriate commercial formulations and application methods to maximize the potential use of biocontrol agents [6].

Several mechanisms have been proposed to explain the antifungal activity of biocontrol agents. Wound colonization and nutrient competition appear as the primary mechanisms [7–11]. Other attributes of yeast that have been associated with their biocontrol activity include the production of antifungal compounds (lytic enzymes, killer toxins, peptides and antibiotic metabolites) [6,12–18]. The most thoroughly studied example is farnesol from *Candida albicans* [19], which can inhibit various bacteria and fungus [20]. Production of antimicrobial compounds is not restricted to *Candida*; they can also be found in other yeast genera: *Cryptococcus*, *Saccharomyces*, *Hanseniaspora*, *Hansenula*, *Kluyveromyces*, *Pichia*, *Rhodotorula*, *Tilletiopsis* and *Meyerozyma* [11,21,22].

Recently, several studies have focused on antifungal compounds from natural sources as an effective alternative to chemical preservatives, e.g., phenylacetic acid (PAA), phenyllactic acid (PLA) and phenylethanol (PEA). PEA, a colorless liquid with a rose-like odour, occurs widely in nature, including in a variety of essential oils extracted from rose, jasmine, carnation and, hyacinths [23]. Greater attention has been paid to studying the antimicrobial properties of PEA [24–27], and in addition screening of yeasts for the production of PEA for natural products in the cosmetic and food industry [28,29].

In spite of the information available in literature, few extensive isolation studies of antibiotic antifungal compounds from antagonistic yeast are available. The objective of this study is to identify and characterize of antifungal compounds from the antagonistic yeast strain *K. apiculata* 34*–*9, and study their fungistatic activities.

## Results

### Extraction of antifungal substances produced by *K. apiculata*

**Figure 1:**
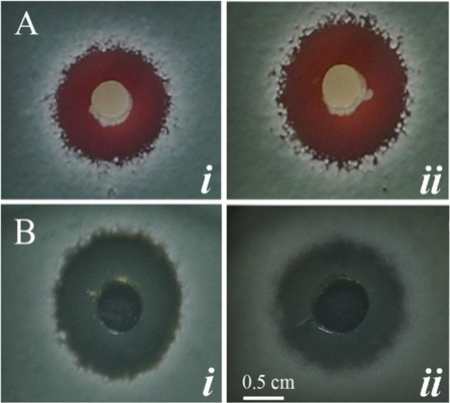
**Effect of** ***K. apiculata*** **and the (ether) extract on** ***Penicillium*** **molds. A**: The agar diffusion assay was performed of *K. apiculata* against *P. italicum* (i) and *P. digitatum* (ii) after 48 h co-cultured at 28°C; **B**: *P. italicum* (i) and *P. digitatum* (ii) incubated for 48 h at 28°C after addition of 10 μL of the extract.

*In vitro*, *K. apiculata* 34–9 showed antagonistic properties against *P. digitatum* and *P. italicum* (Figure 1A) in PDA medium. Meanwhile, antifungal substances were efficiently extracted by ether from both the cell-free culture and cells of *K. apiculata* (Figure 1B).

**Figure 2:**
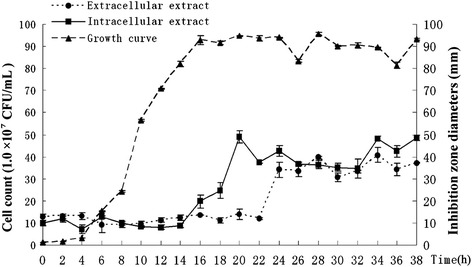
**Relationship of population dynamics of** ***K. apiculata*** **to intra/extra-cellular antifungal activity.** Effect of the number of *K. apiculata* on antifungal compounds secretion was investigated by statistic correlation between the cells number and antifungal activity that was extracted from the cell-free culture (extracellular) and cells of *K. apiculata* (intracellular) respectively. The assays were performed in 50-mL BSM broth at 28°C with 1.0 × 10^8^ cells/mL of *K. apiculata* initially. Samples were analyzed for the number of *K. apiculata*, extra- and intracellular activity at intervals of 2 h. Antifungal effects were recorded with inhibition diameter (mm) after 48 h culture at 28°C in PDA medium (2.0 × 10^5^ spore/mL *P. italicum* spores).

Antifungal activity was not affected by trypase, proteinase K (100 μg/ml, 37°C for 60 min), or high temperature (140°C for 10 min) treatments, but it was sensitive to alkaline pH (data not shown). The activity was stable at pH values between 2.5 and 5.5, but it rapidly decreased between 5.5 and 11.0. A peak of inhibition was observed at the end of log phase (20 h), which is produced by intracellular extraction, and four hours later (24 h) the same peak is produced by extracellular extraction and four hours later (28 h) there was another little increase (Figure 2). The result showed four hours delay between antifungal compound biosynthesis and secretion.

### Purification and identification of antifungal substances

**Figure 3:**
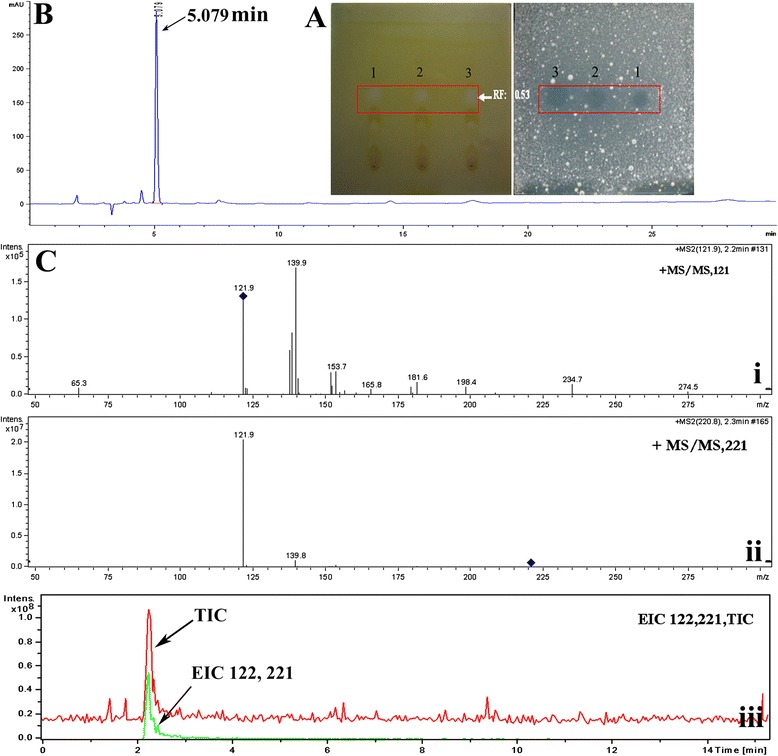
**Purification of the antifungal compound. A**: Silica plate TLC purification. The TLC separation was performed with petroleum ether: ethyl acetate (4:1, v/v) and visualized using iodine vapor. Then compounds in the TLC were directly transferred to PDA medium (mixed with 2.0 × 10^5^ spore/mL *P. italicum* spores) by blotting. The spot of 1, 2 and 3 were antifungal compound (white) in TLC (RF:0.53), its corresponding antifungal spots in PDA. **B**: semi-preparative HPLC purification. The HPLC were performed with C_18_ reversed-phase column, 2:3 methanol-H_2_O (containing 0.1% acetic acid), and 210 nm detection **C**: LC-MS analysis. The LC-MS was analyzed with C_18_ reversed-phase column, ion source temperature, gas temperature 200°C, nebulizer 15 psi and HV capillary 3500 V. (i) 220.8 Tandem MS Chromatogram, (ii) 121.9 tandem MS chromatogram, (iii) Total ion chromatogram (TIL, green) and extracted Ion Chromatogram (EIC, red) for 122 and 221.

The raw extract was first purified by thin-layer chromatography (TLC) eluting with ether and benzene (1:1, v/v). The retention factor (R_f_) value of active fractions was 0.74. We collected these active fractions and further separated them by petroleum ether and ethyl acetate (4:1, v/v). Iodine vapor showed that only a white spot (R_f_ = 0.53) on a light brown background had antifungal activity (Figure 3A). The white spot was collected for the further purification. HPLC revealed that retention time (RT) of this antifungal compound was 5.079 min (Figure 3B).

**Figure 4:**
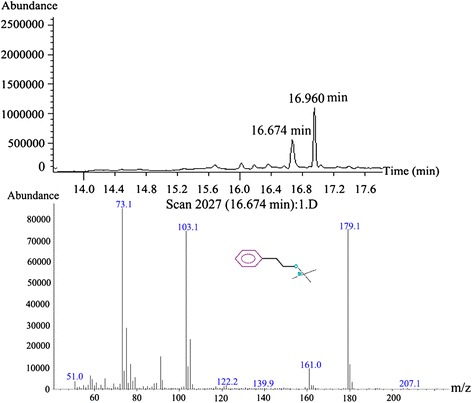
**Identification of the structure of antifungal compound by GC-MS.** Purified sample were analyzed by GC-MS with HP-5MS capillary column chromatography after derivatized with N,O-Bis(trimethylsilyl) trifluoroacetamide. Operating conditions: carrier gas (helium) 1 mL/min, split ratio 50:1, injector temperature and interface temperature 280°C, and oven temperature programmed at 40°C (2 min) and ramped to 280°C at 5°C/min, 70 eV. 16.674 min: PEA (Silane, trimethyl(2-phenylethoxy)); 16.960 min: glycerol (Trimethylsilyl ether of glycerol).

The collected sample from semi-preparative HPLC was analysed by LC-MS. Through the exploration of conditions, the purified sample indicated prominent *m/z* ions at 122 and 221, and their extracted ion chromatogram (EIC) were in keeping with the peak of antifungal compound of LC-MS total ion chromatogram (TIL) and DAD spectrogram. The MS/MS revealed that *m*/*z* 122 and 221 derived from the same compound (Figure 3C). The sample was then analyzed by GC-EI-MS. Consequently, this antifungal compound was determined to be 2-phenylethanol (PEA; RT = 16.674 min) (Figure 4). In addition, other compounds, including 2-phenylacetic acid (PAA; RT = 17.338 min), phenyllactate (RT = 17.961 min), and phenylpyruvate (RT = 19.243 min), were identified in the raw extract, revealing antifungal substances produced by *K. apiculata* were closely related to the L-phenylalanine (L-Phe) metabolic pathway.

**Figure 5:**
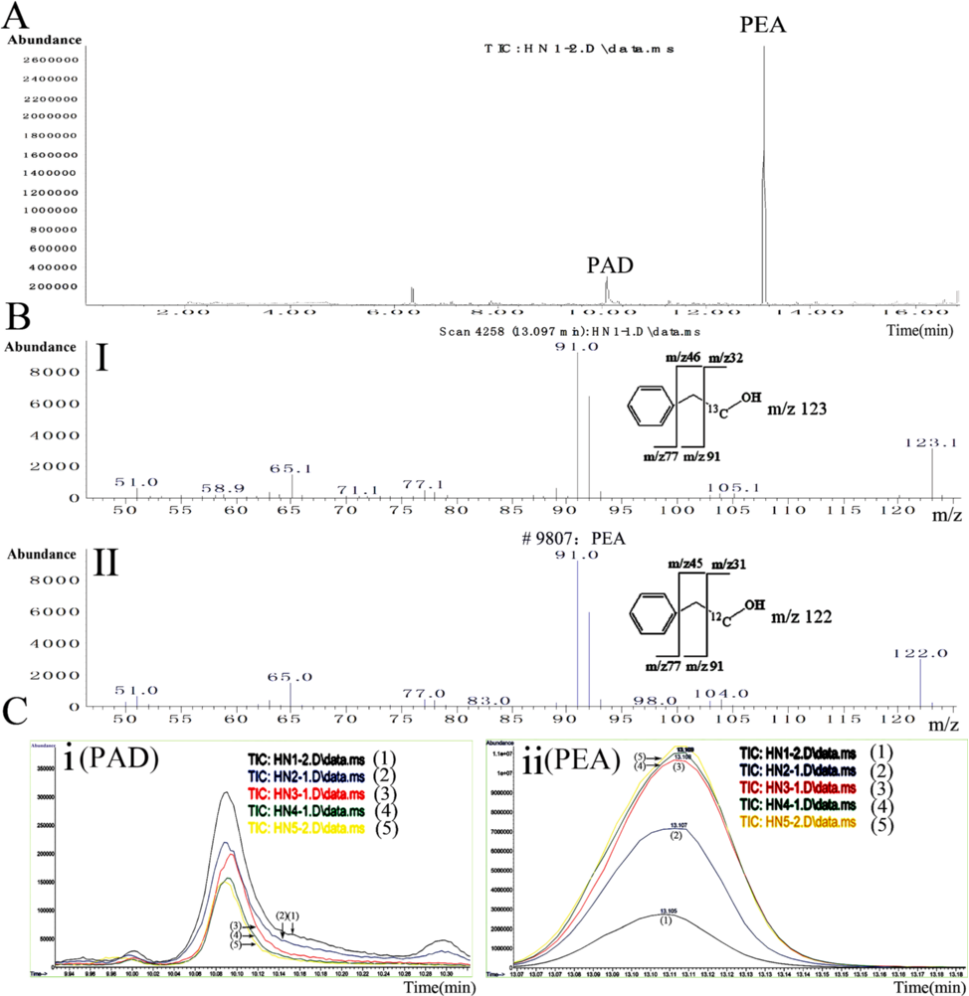
**L-Phe metabolic pathway analysis of** ***K. apiculata*** **by [2-** ^**13**^ **C] L-Phe labeling using GC-MS. A**: Total ion chromatograph (TIC) of *K. apiculata*. **(B-I)** 13.097 min MS and **(B-II)** PEA standard MS. **(C)** Total ion chromatograph (TIC) of 2-phenylacetaldehyde (PAD; 10.09 min) and 2-phenylethanol (PEA;13.10 min) for different culture periods in dextrose minimal medium with [2-^13^C] L-Phe as the sole nitrogen source (0.76% YNB, 2% dextrose, 2% [2-^13^C] L-Phe). (1), (2), (3), (4) and (5) were 3 h, 6 h, 12 h, 24 h and 48 h analysis results after inoculation, respectively.

L-Phe metabolic pathway of *K. apiculata* was analyzed using [2-^13^C] isotope labeling of L-Phe. Figure 5 showed that the largest signal was ^13^C-labeled PEA, followed by ^13^C-labeled 2-phenylacetaldehyde (PAD), PAA and phenylpyruvate. The concentration of PEA in culture increased with time (Figure 5C-ii), whereas ^13^C-labeled PAD decreased with the time (Figure 5C-i). This confirmed that PEA was produced via L-Phe, and that PAD is an intermediate product of PEA [30].

### Antifungal activity of PEA and PAA

**Figure 6:**
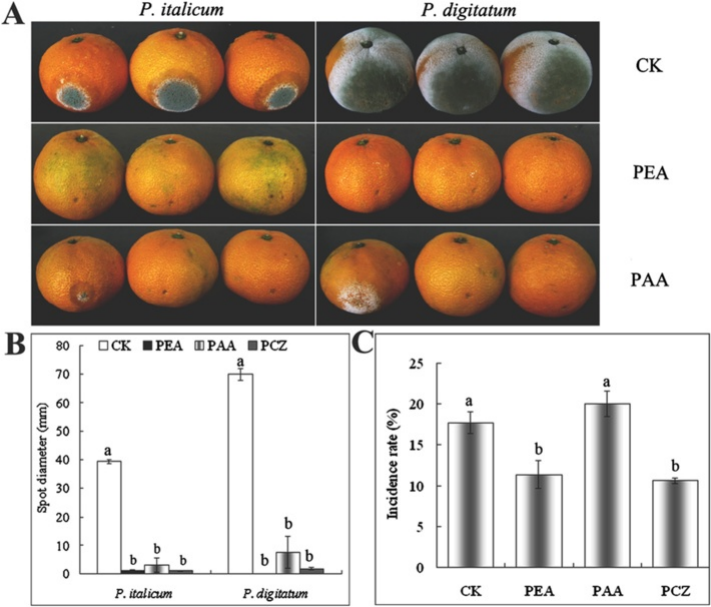
**Effect of 2-phenylethanol (PEA) and phenylacetic acid (PAA) to** ***Penicillium*** **molds. A** and **B**: Fruit inoculation tests (means ± s.d.; Duncan’s test p ≤ 0.05); **C**: Fruit storage test (means ± s.d.; Duncan’s test p ≤ 0.05). PEA (1.5 μL/mL), 45% prochloraz (PCZ) (1500 × dilute), PAA (1 mM).

Growth of blue and green molds was inhibited *in vitro* and *in vivo* by 10 μL of 1.5 μL/mL PEA. In fruit storage assay, 1.5 μL/mL PEA also effectively protected citrus from infection by susceptible strains of green and blue molds (Figure 6A,B), while not affecting fruit quality factors such as ascorbic acid, soluble solid contents, and titratable acid (data not shown). The incidence of blue and gray molds for PEA treatment was 11.3% after 110 d, which was similar to that achieved with prochloraz (10.7%) (Figure 6C).

We also analyzed the antifungal activity of the L-Phe metabolite, PAA, which is known to possess antifungal properties [31]. We found that PAA inhibited the pathogenic fungus *in vitro* and *in vivo*, but it did not effectively control the occurrence of disease in storage (Figure 6C). This result further confirmed PEA as the main antifungal compound of *K. apiculata*.

### Effect of PEA on fungal cells

**Figure 7:**
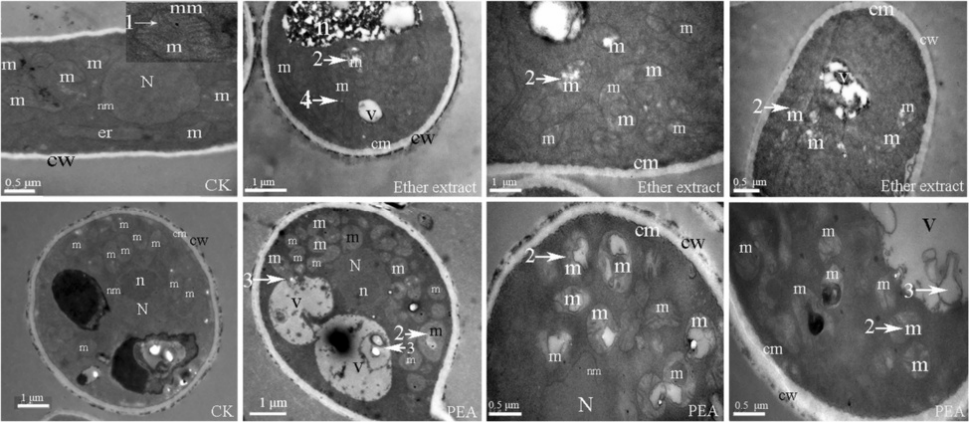
**Effect of the extract and 2-phenylethanol (PEA) on subcellular components of** ***P. italicum*** **.** *P. italicum* was cultured in PDA liquid medium for 8 h, the extract (1000 × dilute) or 1.5 μL/mL PEA were added to medium and further with 2 h culture*.* (m) mitochondrion, (N) nucleus, (n) nucleolus, (cw) cell wall, (cm) cell membrane, (er) endoplasmic reticulum, white arrow (1) mitochondrialcristae, (2) mitochondria abnormalities and vacuolation, (3) organelles degradation, (4) leakage of the outer membrane of mitochondria.

Transmission electron microscopy (TEM) showed that the action of the raw extract (1000 × dilute for 2 h) on ultra-cellular *P. italicum* was closely associated with mitochondrial abnormalities (Figure 7), including degraded and disorganized cristae, leakage of the outer membrane, and massive mitochondrial vacuolation. Meanwhile, PEA (1.5 μL/mL for 2 h) induced hyphal cells with abnormal subcellular morphology (Figure 7). The prominent features were massive mitochondrial vacuolation and vacuole mediated organelle degradation.

**Figure 8:**
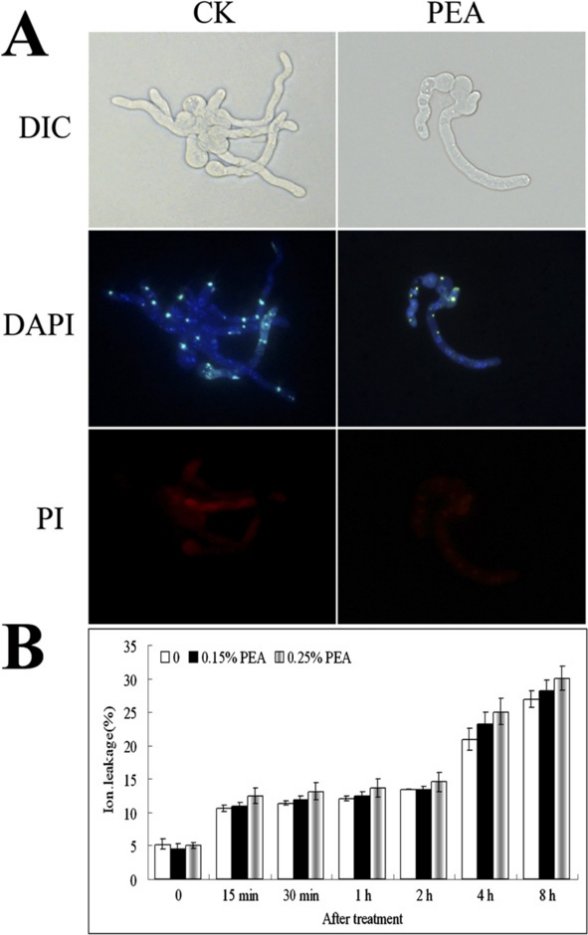
**Effect of PEA on hyphal cytomembranes. (A)** DAPI and PI staining analysis. The *P. italicum* hyphal cells were treated with 1.5 μL/mL PEA for additional 2 h. The cultures were fixed and stained with DAPI and PI. **(B)** Ion leakage analysis (means ± s.d.; Duncan’s test p ≤ 0.05).

To test whether PEA’s inhibition of fungal cells was associated with a change in the permeability of the cytoplasmic membrane, 4′,6-diamidino-2-phenylindole (DAPI) and propidium iodide (PI) staining and membrane electric conductivity were performed (Figure 8). PI is a membrane-impermeable stain in normal healthy cells, but it readily penetrates the membranes of dead cells. As a control, no PI staining in the nucleus was detected in the strain with or without PEA treatment (Figure 8A), consistent with results on conductivity, suggesting that the hyphae were intact (Figure 8B). Similar result was found in hyphae of *Aspergillus flavus* in the presence of PEA [32].

### RNA-Seq profiling of the response of *P. italicum* to PEA

Regulation of gene expression was investigated using comparative RNA-Seq profiling analysis. Samples of 0 h (CK), 1 h (PEA1) and 3 h (PEA3) treatment were used for the construction of RNA-Seq libraries. After removal of reads with adaptors, unknown bases, low-quality reads (leaving tags of 49 nt long), there were total of 11,605,180, 11,611,106, and 11,672,232 successful sequences (clean reads), produced by PEA1, PEA3, and CK, respectively. The distribution of total clean tags was quite similar at PEA1, PEA3, and CK (Additional file 1).

**Figure 9:**
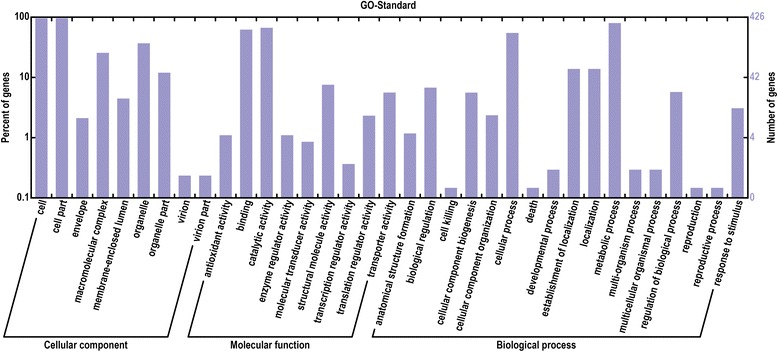
**Functional categorization of the genes with significant transcriptional changes between the PEA treatment and the control type.** The total same changed genes (1304) were categorized based on Gene Ontology (GO) annotation. The proportion of each category is displayed based on: Biological process; Molecular function; Cellular component.

Clean reads were mapped to reference sequences using SOAPaligner/soap2 [33], and mismatches of no more than 2 bases were allowed in the alignment. A total of mapped genes in PEA1, PEA3, and CK were 6905, 6946, and 7051 of the reference genes (Additional file 2). Analysis of differential expression at PEA1 and PEA3 compared to CK revealed 861 and 1160 up-regulated genes, 1072 and 749 down-regulated genes, respectively. Of these, 654 and 650 genes (68.3% of total) share the same up- and down-regulation expression pattern in PEA1 and PEA3. Gene ontology categories were assigned to the 1304 genes with significantly differential expression using the Blast2GO program (http://www.blast2go.org) to evaluate the potential functions of genes that showed significant transcriptional differences between CK and PEA treatment (Figure 9). Seventeen gene categories were defined. Major categories were metabolism (543), cellular (372), localization (94), and regulation (39). The significant enrichment categories classified on the basis of molecular function were catalytic activity (547), binding (510), structural molecule activity (62) and transporter activity (46). The genes were classified on the basis of cellular compound into cell (409), cell part (409), organelle (157), macromolecular complex (109), organelle part (51), membrane-enclosed lumen (19), envelop (9), virion (1) and virion part (1). Among them, membrane-enclosed lumen, macromolecular complex and organelle were major subcellular organelles that responded to PEA stress.

The PEA-responsive genes were further assessed using KEGG pathway analysis. A total of 17 different metabolic pathways were found with more than 3 affiliated genes, of which some were consistent with biological processes that were already identified by GO analysis. The most represented pathways are listed in Table 1. The prominent related pathways were ribosome, amino acids biosynthesis, aminoacyl-tRNA biosynthesis, cell cycle, protein processing in endoplasmic reticulum, RNA polymerase, and DNA replication. Data indicated that ribosome, endoplasmic reticulum and nucleus were the major subcellular organelles in response to PEA, which is in accord with TEM data and the physiological indices.

**Table 1:** **Enrichment pathway analysis of DEGs in** ***P. italicum***

**Pathway**	**CK vs. PEA1** ^**a**^	**CK vs. PEA3** ^**b**^	**Common DGE** ^**c**^	**Pathway ID** ^**d**^
	**(1294** ^**e**^ **) (up-regulated)**	**(1291) (up-regulated)**	**(884) (up-regulated)**	
Ribosome	101 (0)	83 (4)	79 (0)	ko03010
Val, Leu, and Ile biosynthesis	24 (4)	22 (4)	19 (3)	ko00290
Phe, Tyr, and Trp biosynthesis	19 (2)	23 (7)	17 (2)	ko00400
Aminoacyl-tRNA biosynthesis	26 (2)	23 (4)	20 (2)	ko00970
Cell cycle	48 (4)	45 (9)	33 (3)	ko04111
Protein processing in endoplasmic reticulum	39 (19)	32 (26)	19(14)	ko04141
RNA polymerase	13 (0)	8 (2)	6 (0)	ko03020
DNA replication	22 (1)	14 (2)	11 (1)	ko03030
Phosphatidylinositol signaling system	13(12)	11 (11)	8 (8)	ko04070
Meiosis	46 (9)	47 (17)	34 (8)	ko04113
Phagosome	10 (5)	11 (8)	6 (3)	ko04145
Endocytosis	13 (9)	16 (14)	7 (6)	ko04144
Phenylalanine metabolism	15 (5)	16 (8)	11 (5)	ko00360
Proteasome	3 (3)	17 (17)	3 (3)	ko03050
Fatty acid metabolism	28 (25)	36 (33)	20 (15)	ko00071
Peroxisome	24 (15)	35 (27)	19 (14)	ko04146
Regulation of autophagy	9 (8)	5 (4)	4 (4)	ko04140

Pathway enrichment of DEGs was analysis by KEGG annotation (Q-value ≤0.05). ^a^PEA treated for 1 h compared to control, ^b^PEA treated for 3 h compared to control; ^c^The same corresponding change of PEA treated for 1 h and 3 h compared to control; ^d^Pathway ID in KEGG; ^e^Total number of DEGs with pathway annotation.

**Figure 10:**
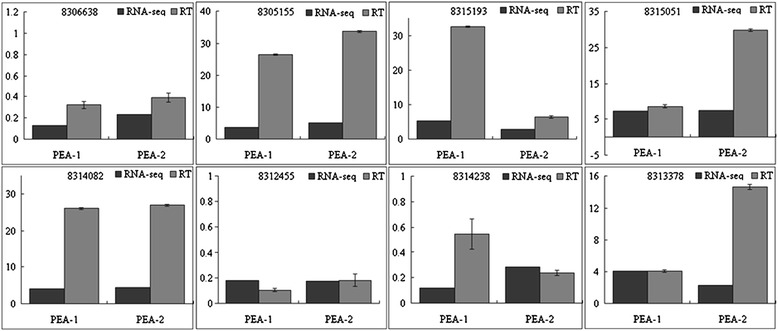
**Real-time quantitative RT-PCR confirmation of the differentially expressed genes between the PEA treatment and the control.** The results were showed by relative transcript times, which acquired by comparison of the transcript abundance of PEA treatment with control. The transcript abundance from RNA-seq data is shown by black columns; Relative transcript levels are calculated by real-time PCR (grey columns) with β-tubulin as the standard (means ± s.d.).

To confirm that the DEGs identified by deep sequencing were indeed differentially expressed, a total of 8 genes were chosen for confirmation in a biologically independent experiment using qRT-PCR, including ribosome, autophagy, proteasome and fatty acid synthesis-related genes, which were detected in the transcriptome and bioinformatic analyses. The relative transcript abundance patterns for the CK and PEA treatment were compared using the transcriptome data. The results of qRT-PCR revealed similar expression patterns as the Illumina sequencing despite some quantitative differences in the expression levels (Figure 10).

## Discussion

Various mechanisms have been proposed to explain the biocontrol of antagonistic yeast to fungal pathogens. Prominent among these is the suggestion that yeast competes with pathogens for nutrients and space [7]. As the safest microbes, yeasts are common on the surfaces of fruits and vegetables, which have used in food preparation for millennia. Previous reports suggest that the antagonistic yeast usually does not depend on the production of antibiotics, but rather on their ability to colonize and grow rapidly in surface wounds [5,34]. Here, we identified an antifungal compound PEA from *K. apiculata* 34–9 that produced by Ehrlich pathway from L-Phe, and their potential application in the field of citrus postharvest pathology is unexplored.

As an aromatic alcohol, PEA has been approved for use to modify certain flavor compositions of foods in the USA. The acute toxicity LD50 (1700 mg/kg for rat) of PEA was classified as low toxicity (24,28). It inhibits a range of bacteria, such as *Bacillus subtilis*, *Ralstonia solanacearum* and *Escherichia coli* [23,24]. In *E. coli,* PEA appears to inhibit the syntheses of DNA [35], and an increase in membrane fluidity caused by PEA has been demonstrated in *Myxococcus xanthus* [36]. Also, PEA inhibits synthesis of macromolecules and phospholipids metabolism [37,38]. Lester [25] showed that PEA exhibited activity *in vitro* against fungi *Neurospora crassa*, and inhibition of growth and of the syntheses of RNA, DNA and protein. Furthermore, PEA inhibits RNA, DNA, protein and aminoimidazole ribotide syntheses, cytoplasmic respiratory, and glucose uptake and incorporation in yeast [39]. Zhu et al. [23] inferred that PEA competes for attachment on the active site of the enzyme with L-3,4-dihydroxyphenylalanine due to its –OH group.

Although the mode of action of PEA on bacteria and fungi has been described, our study represents the first comprehensive transcriptome study of the inhibition process. Illumina sequencing was used to monitor the global transcriptional change in the PEA treatment compared with the control, and 1304 differentially expressed genes that were induced or repressed by more than two fold at different treatment times, were identified. RNA-Seq data showed that the action of PEA on *P. italicum* resembles its effect on *N. crassa* and yeast; ribosome, mitochondrion, macromolecular complex, endoplasmic reticulum and nucleus were the major subcellular organelles in response to PEA.

A number of new genes possibly related with the inhibition process were found in this study. Functional category analysis revealed that a number of important pathways may work collaboratively in inhibiting the fungal cell growth. The first noticeable pathway is the amino acid and protein biosynthesis pathways which exist in the plastids. 97 out of 99 DGEs of ribosome and aminoacyl-tRNA synthetases (AARSs), and 31 out of 36 DGEs of amino acid biosynthesis were down-regulated (Table 1). Based on these data, it can be speculated that the inhibition of the amino acids and protein biosynthesis resulted in inhibition. The structure of PEA and RNA-Seq data from PEA-inhibited cultures lead us to suppose that the PEA might compete for attachment on the active site of fungi phenylalanyl-tRNA synthetase (PheRS) by the formation of a stable tRNA^Phe^-PEA [40,41], thus inhibiting protein synthesis, RNA transcription and energy demanding processes.

The second group of PEA-responsive pathways includes DNA replication, meiosis and cell cycle pathways. Most of these genes also exhibited down-regulation pattern in PEA treatment. The induction of genes associated with cell cycle is consistent with the GO results that indicate that large numbers of genes are located in nucleus; and is in line with our physiological analysis that the nucleus is one of the major subcellular organelles in response to PEA.

The third group of metabolite pathways represents those related with cell death including phagosome, proteasome, peroxisome and regulation of autophagy. Most of these genes were up-regulated indicated that PEA induced fungal cells autophagy or programmed cell death.

Our study also showed that PEA and PAA were identified in the raw extract, and both of them are known to have antimicrobial properties. Both PEA and PAA inhibited citrus green and blue molds *in vitro* and *in vivo*. However, PAA was ineffective in fruit storage. Fungus pathogens suppress host cell defense responses by acidification of the fruit with organic acids, such as citric and gluconic [42–45]. Maybe, PAA causes low pH in fruit tissue.

## Conclusions

We have identified an antifungal compound from biocontrol agent *K. apiculata* 34–9 and provided a global picture of the gene expression changes in a PEA treatment comparing with control type. The interpretation of the Illumina sequencing data uncovered a large number of genes with previously not known to be involved in the inhibition process. Functional categorization of the differentially expressed genes showed that a number of important pathways, including amino acids and protein biosynthesis, cell cycle and cell death cross communicated and worked collaboratively in inhibiting phytopathogenic fungi. This study provided new insight into the mode of action of biocontrol yeast agents in controlling postharvest pathogenic fungi.

## Methods

### Antagonist and fungal pathogens

Strain of *K. apiculata* 34–9 was isolated from the rhizosphere soil [46]. The strain was grown in BSM (20% bean sprout extract, 5% dextrose, 2% agar), YPD (1% yeast extract, 2% peptone, 2% dextrose, 2% agar), and minimal medium (0.17% yeast nitrogen base without amino acids and ammonium sulfate (YNB; Difco), 2% dextrose, 2% L-phenylalanine). The molds of *P. digitatum* and *P. italicum* were used as test fungus, which were cultured in PDA (20% potato extract, 2% dextrose, 2% agar).

### Collection and extraction of antifungal compounds

*K. apiculata* was grown in BSM broth at 28°C for 48 hour with shaking at 200 rpm. After incubation, cells were removed by centrifugation at 8000 × *g* for 10 min and sterile filtered (0.45 μm). The cell-free culture was extracted using a series of organic solvents (1:1; v/v), including cyclohexane, petroleum ether, benzene, chloroform, ether, acetic ether, and *n*-butyl alcohol. Each extraction was performed twice and extracts were pooled and concentrated using a rotary evaporation (Laborota model 4010, Heidolph, Germany) leaving a yellow oil as the product, which was used directly for *in vitro* assaying of antifungal activity.

### Citrus fruit

Navel orange fruit (*Citrus sinensis* L. Osbeck) were harvested from the orchard (Yichang, Hubei, China) for *in vivo* and fruit storage assays. Fruits without physical injuries and infections were selected based on uniformity in size. Prior to use, fruits were disinfected with 2% (*v/v*) NaOCl solutions for 2 min, rinsed with tap water and air-dried.

### Antagonism assays

*In vitro*, *in vivo* and fruit storage assaying of antifungal activity were conducted with two biological replicates as described previously [46].

Briefly, *in vitro* antifungal activity was carried out using a disc-diffusion method. Petri plates were prepared with 15 mL of PDA medium. PEA (1.5 μL/mL, 10 μL), PAA (1 mM, 10 μL) and sterile distilled water (10 μL) were pipetted into the 5 mm (diameter) holes of agar punched in the agar plates with inoculum of 1.0 × 10^5^ conidia/mL of *Penicillium* suspension.

*In vivo*, orange fruits were wounded with a bodkin to a 5 mm depth with two wounds per orange. PEA (1.5 μL/mL, 10 μL), 45% prochloraz (PCZ) (1500 × dilute, 10 μL), PAA (1 mM, 10 μL) and sterile distilled water were pipetted onto each wound after inoculation of 10 μL of a 5.0 × 10^5^ conidia/mL of *Penicillium* suspension. After treatment, the fruits were placed in plastic chambers to maintain a high relative humidity of approximately 95% and maintained at 25°C. The percentage of disease inhibition was determined by measuring the diameter of the lesions produced by the fungus in each fruit wound after five days.

For fruit storage assay, individual fruits were dipped for 5 min in PEA (1.5 μL/mL), 45% prochloraz (PCZ) (1500 × dilute), PAA (1 mM) and sterile distilled water (control). After air dry, fruits were directly stored in a ventilated warehouse (about 5–10°C) for natural storage test. Visual decay was evaluated after 3 months of storage. Each treatment was replicated three times with 100 fruit samples per replicate.

### Influence of *K. apiculata* growth on production of antifungal compounds

Effect of the number of *K. apiculata* on antifungal compounds secretion was investigated comparing the cells number and antifungal activity that extracted respectively from the cell-free culture (extracellular) and cells of *K. apiculata* (intracellular). The assays were performed in 50-mL BSM broth at 28°C with 1.0 × 10^7^ cells/mL of *K. apiculata* initially. Samples were analyzed the number of *K. apiculata*, extra- and intracellular activity at intervals of 2 hour.

Extracellular extraction of antifungal compound was performed as described above. For intracellular cells, yeast cells were collected by centrifugation at 8000 × *g* for 10 min and washed twice. The cells were ground into a powder in liquid nitrogen, and then added sterile distilled water to original cultivated volume for extraction.

### Purification and identification of antifungal compounds

The (ether) extract was analyzed by thin-layer chromatography (TLC; silica gel G plates) in the following solvent 50% ether, and 50% benzene. The second separation was performed with 75% petroleum ether, and 25% ethyl acetate. The plates were visualized using I_2_ vapor. The active fractions were eluted with methanol and concentrated using rotary evaporation and pooled. These were then purified by C_18_ reverse-phase HPLC (Agilent 1200; Santa Clara, CA, USA) employing a C_18_ column (4.6 mm × 250 mm; 5-μm; Elite, Dalian, China). The column was eluted at 1 mL/min, with an optimized concentration using 40% methanol and 60% H_2_O (0.1% (*v/v*) acetic acid). Chromatograms were scanned at 210 nm. The antifungal activity was examined for each substance collected in each fraction.

Separation of the purified compounds for mass spectrometry analysis was achieved with an Agilent 1100 series LC/MSD Trap. The separation was carried out with C_18_ column (4.6 mm × 150 mm; 5 μm; Agilent). Mass spectrometry operating were ion source temperature and gas temperature 200°C at a drying gas flow 5.00 L/min, nebulizer 15 psi and HV capillary 3500 V. Full scan spectra from *m/z* 50 to 1000 in both positive and negative ion modes were recorded.

GC-MS analysis of the purified compounds was performed by Agilent 6890A/5975C and equipped with HP-5MS (30 m × 0.25 mm; 0.25 μm; Agilent) capillary column. Helium was used as carrier gas at a flow rate of 1 mL/min and split ratio 50:1. The injector and interface temperature was set to 280°C. The analysis was performed under the following temperature program: 2 min of isothermal heating at 40°C and then ramped to 280°C at 5°C/min. 70 eV of electron energy was used for simple ionization. Mass spectra were scanned from *m/z* 50 to 1000. In addition, the purified compounds were also analyzed by GC-MS after reacted with N,O-Bis(trimethylsilyl) trifluoroacetamide (BSTFA, Fluka) at 100°C for 15 min.

### Microscopic observation of the fungus

The fungus for the transmission electron microscope (TEM) samples were harvested and fixed overnight in 2.5% glutaraldehyde at room temperature. After washing with in a 1 M PBS (pH 7.2), the specimens were postfixed in 1% osmium tetroxide buffer for 2 h, washed again, and then dehydrated by a graded acetone series (30–100%). The samples were embedded in Spurr’s low-viscosity resin for 48 h at 45°C. Semithin and ultrathin sections (40–60 nm) were cut with a Leica Ultracut RM2265 (Leica, Vienna, Austria), mounted on regular hexagonal copper grids, stained with lead citrate (10 min), washed three times with ddH_2_O, stained with uranyl acetate for 30 min, washed again, and examined with a JEOL H-7650 TEM (Hitachi High-Technologies Co., Japan).

### *K. apiculata* L-phenylalanine-related metabolite assay

Experiments were performed in minimal medium containing 2% [2-^13^C] L-phenylalanine (L-Phe) (99 atom%; Cambridge Isotope Laboratories, Cambridge, MA) as single nitrogen source. Liquid medium inoculated with 1.0 × 10^7^ cells/mL was grown at 28°C for 3 hour, 6 hour, 12 hour, 24 hour and 48 hour, respectively. The concentrations of the various end products of L-Phe metabolites in culture filtrates were determined by using GC-MS, as described above.

### 4′6-diamidino-2-phenylindole (DAPI) and propidium iodide (PI) staining

Fungal nuclei to be observed by fluorescence microscopy were stained with DAPI and PI. After 12 hour of growth and 1.5 μL/mL PEA treatment for 2 hour, strains were fixed and stained with DAPI and PI as described previously [18]. Conductivity detection was performed as Wang et al. [47].

### *Penicillium* RNA extraction, Illumina sequencing and data analysis

Untreated (CK), and PEA-treated samples (PEA1 and PEA3 refer to 1.5 μL/mL-treated fungus for 1 hour and 3 hour, respectively), were harvested. Total RNA was extracted from *P. italicum* by Trizol (Invitrogen, Carlsbad, CA, USA) following the manufacturer’s instructions. RNA-Seq profiling was performed by Beijing Genomics Institute (Shenzhen, China). Brifly, mRNA was enriched by using oligo(dT) magnetic beads. The fragmentation buffer was added and mRNA was interrupted to approximately 200 bp. The first and second strand cDNA was synthesized by using reverse transcriptase and random hexamer-primer. Double-strand cDNA was purified with QiaQuick PCR extraction kit and washed with EB buffer for end repair and single nucleotide adenine addition. Finally, sequencing adapters were ligated to the fragments. The required fragments were purified by agarose gel electrophoresis and enriched by PCR amplification. The sequences of the library products were analyzed by using an Illumina HiSeq™ 2000.

Clean reads were generated by removing adapter sequences, unknown bases more than 10% and low quality reads. Each tunnel generated 11.6 million reads with a sequencing length of 49 bp. All clean reads were then aligned to reference sequences of *Penicillium chrysogenum* Wisconsin 54–1255 by using SOAPaligner/soap2 [33]. Mismatches no more than 2 bases were allowed in the alignment. The expression level of gene (Additional file 2: Table S1) was calculated by using the RPKM (reads per kb per million reads) method [48]. Differentially expressed genes (DEG; Additional file 2: Table S1) in three samples were analyzed as described [49–52].

### Real-time quantitative RT-PCR (qRT-PCR) verification

Eight genes were chosen for confirmation by qRT-PCR with SYBR Premix Ex Taq™ (Takara, Japan). Primers for the chosen genes were designed with the Primer Express software (Applied Biosystems, USA) and are presented in Additional file 3. qRT-PCR for gene expression analysis was performed on a StepOne Real-time PCR System (Applied Biosystems, USA) using β-tubulin gene as an endogenous control. Briefly, the primers for the target gene and β-tubulin were diluted in the SYBER Mix (Applied Biosystems) and 20 μL of the reaction mix were added to each well. The reactions were performed with an initial incubation at 50°C for 2 min and at 95°C for 1 min followed by 40 cycles of 95°C for 15 s and 60°C for 1 min. The levels of gene expression were analyzed with StepOne Software v2.0. Zero template controls were included for each primer pair. Each PCR reaction was carried out in triplicate, and the data are presented as the means ± SD.

## Additional files

Additional file 1:
**The length distribution of Gene coverage.**
Additional file 2:
**All differential expression between SBS and CK fruit.**
Additional file 3:
**Primers used for real-time quantitative RT-PCR for the verification of RNA-Seq profiling data.**
