# Supplementary figures and images for: Mechanisms of action for 2-phenylethanol isolated from Kloeckera apiculata in control of Penicillium molds of citrus fruits
Source: BMC Microbiol. 2014 Sep 19;14:242. doi: 10.1186/s12866-014-0242-2 (PMC4177429; doi:10.1186/s12866-014-0242-2)

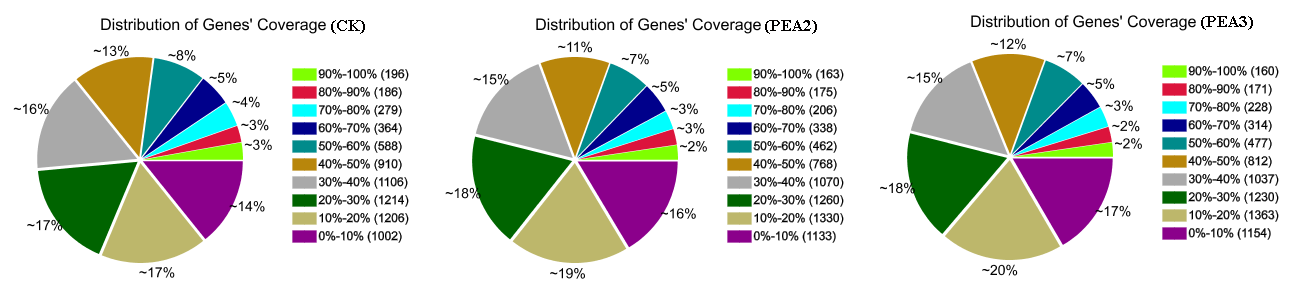

Supplement: Additional file 1: — The length distribution of Gene coverage. [file 12866_2014_242_MOESM1_ESM.tiff]
