# Supplementary material for: Mechanisms of action for 2-phenylethanol isolated from Kloeckera apiculata in control of Penicillium molds of citrus fruits
Source: BMC Microbiol. 2014 Sep 19;14:242. doi: 10.1186/s12866-014-0242-2 (PMC4177429; doi:10.1186/s12866-014-0242-2)
Supplement: Additional file 3: — Primers used for real-time quantitative RT-PCR for the verification of RNA-Seq profiling data. [file 12866_2014_242_MOESM3_ESM.doc]

***Additional file 3 Primers used for real-time quantitative RT-PCR for the verification of RNA-Seq profiling data***

|  | sequences | Related pathway | bp |
| --- | --- | --- | --- |
| 8312455 L | GCTGCCAAGAACCCCCTTA | Ribosome gi|255946966 | 19 |
| 8312455 R | GGATGTCCTGGCCGATACC |  | 19 |
| 8314238 L | TCGGTGAGAACGTCGTGATC | Ribosome gi|255942501 | 20 |
| 8314238 R | TGTGGGCAACAACCTCGAT |  | 19 |
| 8305155 L | CGCGGAGTGCCATGTCA | autophagy gi|255949500 | 17 |
| 8305155 R | ATCCTCGAGCCCTGCATCT |  | 19 |
| 8313378 L | TCCCCTCCCGCCTCAT | autophagy gi|255945087 | 16 |
| 8313378 R | GCATACTGGAGTTCTGCGATTCT |  | 23 |
| 8314082 L | CCCAGCGGCCGTATCTTA | Fatty acid metabolism  gi|255940974 | 18 |
| 8314082 R | CTCCGTGTGCGGGTAGATG |  | 19 |
| 8315051 L | GCGCGAATTTTATAAGCTGGTT | Proteasome  gi|255931117 | 22 |
| 8315051 R | CAGCCAGACCGACGATCAG |  | 19 |
| 8315193 L | TCGAGGAATGGCGAAAGG | Proteasome gi|255947754 | 18 |
| 8315193 R | CTTGCTACCCGAGGTGTAGGAT |  | 22 |
| 8306638 L | TCGAGCAGTCCATTCACCCTAT | Fatty acid synthesis  gi|255934800 | 22 |
| 8306638 R | ACGCCAATCGCCTTGGT |  | 17 |
| β-tubulin L | CTAGGCCAGCGGTGACAAGT |  | 20 |
| β-tubulin R | TGGTACCGGGCTCCAAATC |  | 19 |
